# A new method for acquiring images of meiobenthic images using the FlowCAM

**DOI:** 10.1016/j.mex.2018.10.012

**Published:** 2018-10-23

**Authors:** Tomo Kitahashi, Hiromi Kayama Watanabe, Masashi Tsuchiya, Hideyuki Yamamoto, Hiroyuki Yamamoto

**Affiliations:** a Japan Agency for Marine-Earth Science and Technology (JAMSTEC), 2-15 Natsushima-cho, Yokosuka, 237-0061, Japan; b Am-Lab Inc., Venture Plaza Funabashi 216, 1-17-25 Kitahon-cho, Funabashi, Chiba, 273-0864, Japan

**Keywords:** FlowCAM method for identifying and quantifying meiobenthos, Meiobenthos, FlowCAM, Environmental monitoring, Seasonal variation

## Abstract

The purpose of this study was to develop a new method for investigating sediment-inhabiting meiobenthos using the Flow Cytometer And Microscope (FlowCAM). Meiobenthos are widely recognized as a useful indicator for assessing the effects of anthropogenic and natural disturbances in both shallow and deep ocean ecosystems. These small benthic invertebrates are traditionally investigated by individually counting and identifying specimens under a microscope, which is labor intensive and time consuming. However, FlowCAM, which was originally developed to semiautomatically analyze microplankton, has the potential to resolve these challenges. Meiobenthic specimens were extracted from sediment using the centrifugal separation method and were then pipetted into the FlowCAM system and imaged. The images were then used to classify and count the specimens at high taxonomic levels to verify the effectiveness of this method compared with traditional methods. We found that FlowCAM system:

• Enabled sufficient meiobenthic images to be obtained to allow the identification and classification of specimens at high taxonomic levels.
• Obtained comparable numbers of individuals to traditional methods.
• Has the potential to rapidly process large the volumes of meiobenthos samples that are required when monitoring seasonal and spatial variation in ocean ecosystems and conducting long-term environmental impact assessments.

## Graphical abstract

**Figure:**
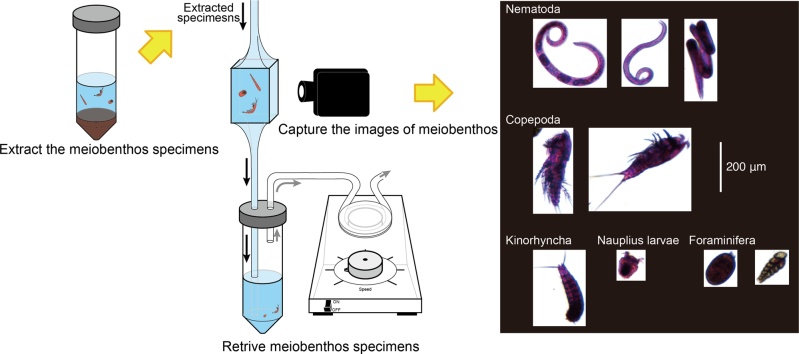

**Specifications Table**

**Table:** 

Subject Area	• *Environmental Science*
More specific subject area	Meiobenthos monitoring
Method name	FlowCAM method for identifying and quantifying meiobenthos

## Method details

Meiobenthos are small benthic invertebrates that pass through a 500–1000 μm sieve and are retained on a 32–63 μm sieve. These animals are important components of deep-sea benthic ecosystems because they are more abundant than larger macro- and megabenthos [1] and have a considerable influence on sediment nutrient cycling and stability, making them good biological indicators of anthropogenic and natural disturbances [2,3]. Nematodes and copepods are the first and second most dominant taxa in deep-sea metazoan meiobenthic assemblages (e.g., [2]), and the latter are more sensitive to environmental stress than nematodes, resulting the nematode-to-copepod ratio (N/C index) tending to increase under polluted conditions. Consequently, this N/C index has been used to measure a range of environmental impacts, including the impacts of pollution [4], anthropogenic activities on sandy beaches [5], the Deepwater Horizon oil spill in the northern Gulf of Mexico in 2010 [6], deep-sea mining for manganese nodules in the central Indian Ocean [7], and fish farming [8].

Traditional methods of investigating meiobenthos include individually counting and identifying small specimens under a microscope. However, this approach is both labor intensive and time consuming. Therefore, alternative methods that allow high volumes of meiobenthic samples to be processed are required, particularly when monitoring spatial and temporal variations in ocean ecosystems or conducting long-term environmental impact assessments, to allow the rapid detection of drastic changes and subsequent environmental monitoring.

The Flow Cytometer And Microscope (FlowCAM) system (Fluid Imaging Technologies, Scarborough, ME, USA) was originally developed to semiautomatically analyze microplankton [9,10] and works by drawing a sample into the flow cell using a pump and obtaining images of the particles in this flow using a digital camera. This system has the potential to resolve the aforementioned challenges in identifying and quantifying meiobenthos. However, meiobenthos cannot be examined in the same manner as plankton using the FlowCAM system due to sediment contamination. Therefore, appropriate modifications are needed to examine meiobenthic assemblages using FlowCAM.

## Problems and solutions when using FlowCAM to investigate meiobenthos

*Problem: Sediment particles block the narrow flow cell*

*Solution: Sieving and separation*

Sediment samples typically contain more sediment particles than meiobenthic specimens resulting in the sediment particles becoming stacked in the flow cell when sediment samples are poured directly into the FlowCAM as well as many more images being required, which increases the volume of data and image-analysis time.

Sieving is an essential process for separating meiobenthic organisms, which are in a specific size range, from bulk sediment samples. The bulk sediment was firstly sieved through 1 mm mesh and the sediments that passed through the sieve were retained on another mesh that was smaller than the size of the low cell to avoid stacking by particles. And then the sediment fraction that passed though the latter mesh was sieved another mesh that was equivalent to the lower size of meiobenthos.

Meiobenthic specimens could be separated from sediment and other debris using a centrifugal method with a high-density solution (e.g., colloidal silica at 1.15–1.18 g/cm^3^) [11,12] to provide suitable specimens for determination by FlowCAM.

When pouring the separated particles were poured into the FlowCAM with water, they moved through the flow line faster than the water due to the difference of the density, resulting in the flow cell becoming clogged with particles, which could stop the FlowCAM from running. This problem was resolved by using a high-density solution (colloidal silica) in place of water, which enabled us to readily adjust the flow speed. Alternatively, high-density solutions that stabilize DNA/RNA (e.g., RNAlater) could be used for downstream genetic analysis.

*Problem: Syringe pump destroys meiobenthic specimens*

*Solution: Use an external pump*

**Fig. 1:**
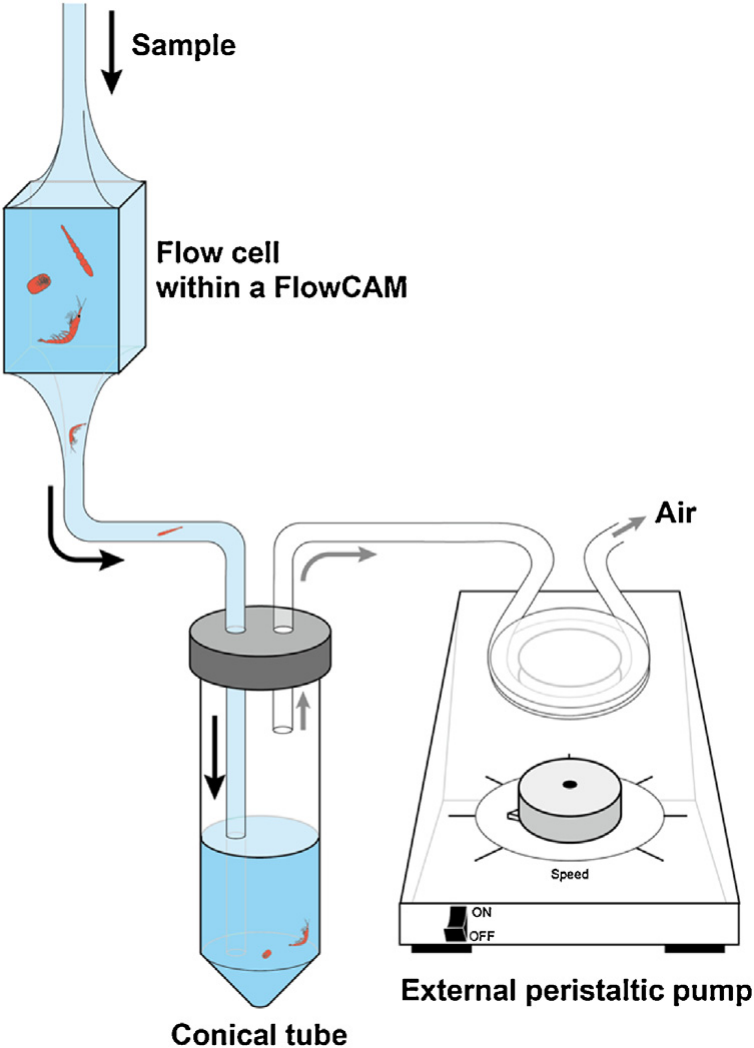
Schematic diagram of the flow line of a FlowCAM system. Black and gray arrows indicate the fluid and air flows, respectively. The fow line was filled with a high-density solution, arranged the specific density of 1.15–1.18 g/cm^3^.

FlowCAM usually transports fluids with an internal syringe pump. However, this type of pump can destroy meiobenthic specimens during retrieval. Therefore, an external peristaltic pump was used and connected to the flow line, as shown in Fig. 1, to allow intact menobenthic specimens to be retrieved. To construct this system, two holes were drilled into the lid of a conical tube or plastic paraffin film (Parafilm, Bemis) was placed over the conical tube and two holes were drilled into this film. Tubes were then inserted into each hole–one connected to the FlowCAM and the other connected to the peristaltic pump, like an aspirator.

*Problem: Sorting the obtained pictures is time consuming*

*Solution: Stain meiobenthic specimens with Rose Bengal*

The organisms were stained red with Rose Bengal to help discriminate them from inorganic particles. This allowed us to sort the organisms by color using a programmed function. The images of red-stained organisms were sorted either first or last according to the red/green ratio using the sort function in *VisualSpreadSheet*, which is the analytical software included with the FlowCAM. Once sorting was completed, the images of organisms could be selected and used to classify and count individuals by taxonomic group.

## Method validation

To verify the effectiveness of this new method for identifying and quantifying meiobenthic organisms, we used it to examine sediment samples collected from four stations established off Otsuchi Bay, northeastern Japan (OT3, 65 m; OT4, 303 m; OT5, 1064 m; OT6, 1677 m water depth) during a cruise of the R.V. *Shinsei Maru*, KS-15-01, in March 2015. Each sample was fixed and preserved in 5% buffered seawater formalin and stained with Rose Bengal (final concentration, 0.05 g/L) until processing in the laboratory. The topmost 0–1 cm layers from a single core from each station was used to validate this novel method.

In the laboratory, the sediment samples were sieved through 1 mm, 250 μm and 63 μm mesh sieves. The fractions that were retained on the 63 μm mesh sieve were then resuspended and centrifuged three times in colloidal silica (Ludox HS-40, Sigma-Aldrich, density, 1.3 g/mL) at 800 × g for 10 min. Observations were made using a portable FlowCAM with a ×4 objective lens and a 300 μm-thick flow cell. The flow line was set up as described above and was filled with colloidal silica (Ludox HS-40) before examination of the samples. The separated samples were gently shaken and suspended in the colloidal silica and were then pipetted into the FlowCAM system. Images of the specimens were captured using FlowCAM’s Auto Image mode at an Auto Image Rate of 20 frames per second. The captured images were processed as previously described using *VisualSpreadSheet* and were then assessed and classified to higher taxonomic levels, such as Nematoda and Copepoda, by the study team.

After examination with FlowCAM, the samples were recollected and reexamined under a binocular stereoscopic microscope for classification and enumeration at higher taxonomic levels. The original conical tubes that contained the samples before they were examined with FlowCAM were also assessed for any remaining samples, and the inflow tube and flow cells of the FlowCAM were washed after each examination and checked for specimens using a stereoscopic microscope. It was found that < 3% of specimens remained in the original conical tubes, inflow tube, and flow cells, indicating that nearly all of the meiobenthic specimens were streamed into the flow cell and recollected after the FlowCAM examinations.

A regression analysis was conducted to assess the relationship between the numbers of individuals observed with microscopy and those observed using the FlowCAM system. All analyses were performed using R, v 3.1.3 [13].

**Fig. 2:**
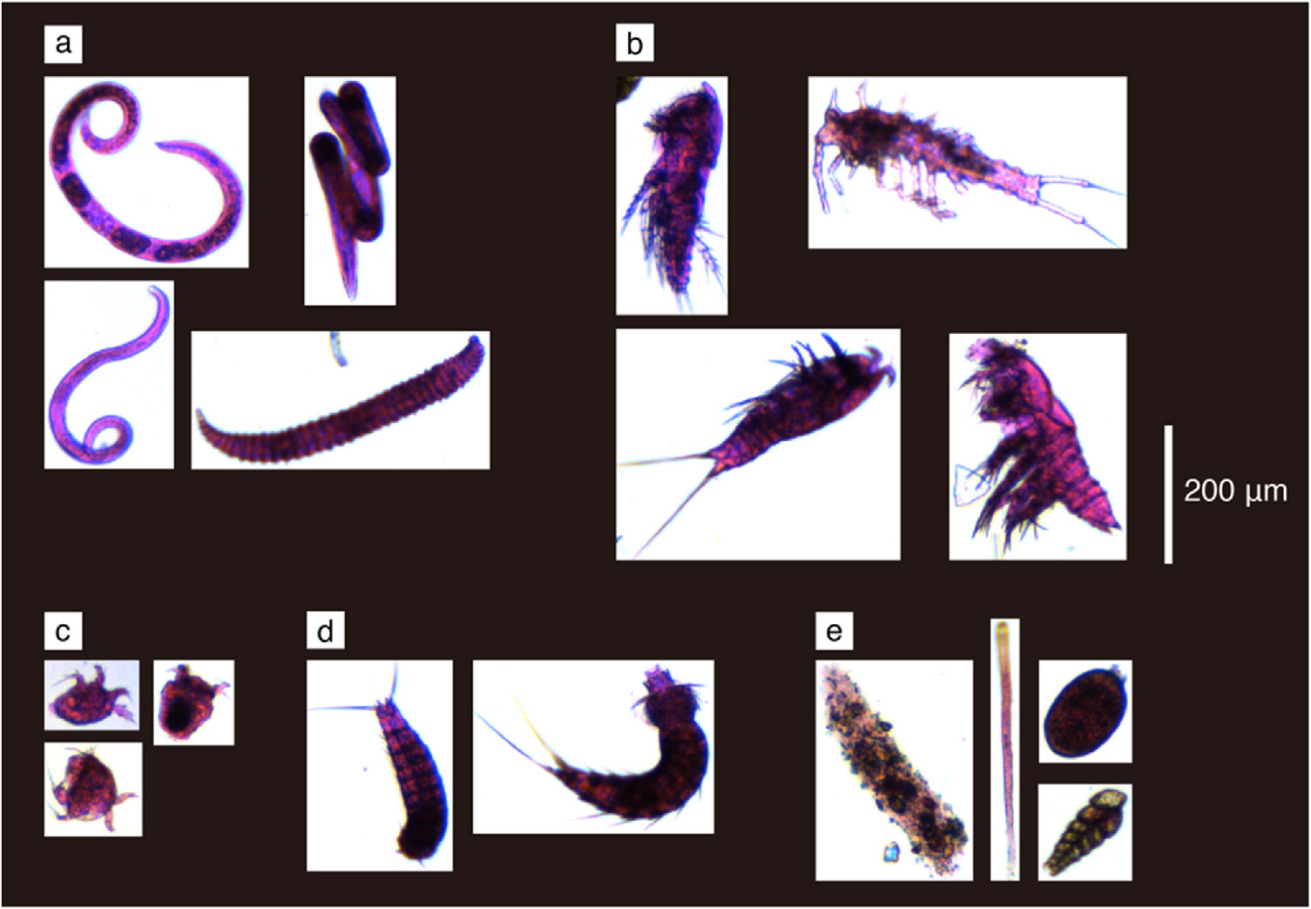
**Meiobenthos images captured using FlowCAM.** Labels indicate the following meiobenthic taxa: a, Nematoda; b, Copepoda; c, Nauplius larvae; d, Kinorhyncha; e, Foraminifera.

**Fig. 3:**
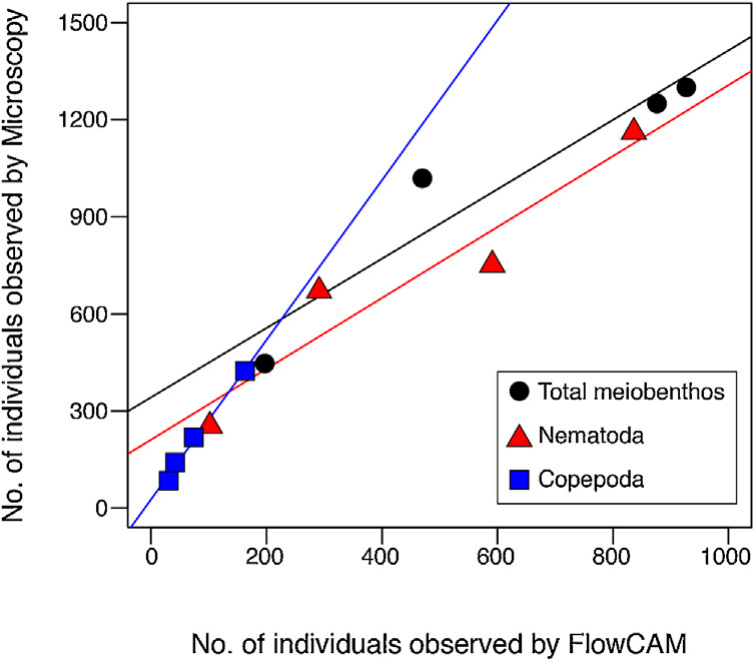
Relationship between the numbers of metazoan meiobenthos observed using FlowCAM and microscopy.

FlowCAM successfully captured the images of the meiobenthic specimens in the samples, including metazoan meiobenthos and protists (mainly, foraminifera; Fig. 2). Comparison of the numbers of individuals observed with FlowCAM and microscopy at each station are provided in Table 1. The FlowCAM imaging efficiencies (i.e., the ratio of the numbers of individuals observed with FlowCAM to microscopy) were 57.9% ± 14.8% for all meiobenthos, 58.5% ± 19.6% for Nematoda, and 34.6% ± 3.7% for Copepoda, and there were significant linear relationships were detected between the numbers of individuals observed with FlowCAM and those with microscopy (*r* = 0.95, *p* < 0.05 for all meiobenthos; *r* = 0.95, *p* < 0.05 for Nematoda; *r* = 1.00, *p* < 0.01 for Copepoda; Fig. 3).

**Table 1:** The numbers of total meiobenthos (a), nematoda (b), and copepoda (c) observed using microscopy and FlowCAM and the imaging efficiencies (ratio of the numbers of individuals observed with FlowCAM to microscopy) at each station.

	OT3	OT4	OT5	OT6
**a) Total meiobenthos**				
Microscopy	1,019	1,250	1,300	447
FlowCAM	470	876	927	197
Imaging efficiency	46.1	70.1	71.3	44.1

**b) Nematoda**				
Microscopy	671	752	1,162	254
FlowCAM	291	591	836	102
Imaging efficiency	43.4	78.6	71.9	40.2

**c) Copepoda**				
Microscopy	219	424	85	141
FlowCAM	74	163	31	42
Imaging efficiency	33.8	38.4	36.5	29.8

A higher ratio of the number of Nematoda to the total meiobenthos was obserbed at OT5 (approximately 90%) compare with the other stations (approximately 50%–70%) using both methods. The study area experienced a large earthquake in March 2011 that may have had different effects at the various stations, resulting in the observed differences in nematode ratios, as reported by studies in adjacent areas [[14], [15], [16]]. Therefore, this finding demonstrates the utility of the FlowCAM method for detecting the effects of natural disturbances.
